# *ABCC6* mutations in pseudoxanthoma elasticum: an update including eight novel ones

**Published:** 2008-01-24

**Authors:** Astrid S. Plomp, Ralph J. Florijn, Jacoline ten Brink, Bruce Castle, Helen Kingston, Ana Martín-Santiago, Theo G.M.F. Gorgels, Paulus T.V.M. de Jong, Arthur A.B. Bergen

**Affiliations:** 1 Department of Clinical and Molecular Ophthalmogenetics, Netherlands Institute for Neuroscience, an institute of the Royal Netherlands Academy of Arts and Sciences, Amsterdam, The Netherlands; 2 Department of Clinical Genetics, Academic Medical Center, Amsterdam, The Netherlands; 3 Wessex Clinical Genetics Service, Princess Anne Hospital, Southampton, United Kingdom; 4 Regional Genetic Service, St. Mary’s Hospital, Manchester, United Kingdom; 5 Department of Pediatric Dermatology, Hospital Son Dureta, Mallorca, Spain; 6 Department of Ophthalmology, Academic Medical Center, Amsterdam, The Netherlands; 7 Institute of Epidemiology and Biostatistics, Erasmus Medical Center Rotterdam, Rotterdam, The Netherlands

## Abstract

**Purpose:**

Pseudoxanthoma elasticum (PXE) is an autosomal recessive disorder of connective tissue, affecting the retina, the skin, and the cardiovascular system. PXE is caused by mutations in *ABCC6*. Up to now, the literature reports that there are 180 different *ABCC6* mutations in PXE. The purpose of this paper is to report eight novel mutations in *ABCC6* and to update the spectrum and frequency of *ABCC6* mutations in PXE patients.

**Methods:**

Eye, skin, and DNA examinations were performed using standard methodologies. We newly investigated the gene in 90 probands by denaturing high-performance liquid chromatography (dHPLC) and direct sequencing. We examined a total of 166 probands.

**Results:**

Eight novel *ABCC6* mutations (c.1685T>C, p.Met562Thr; c.2477T>C, p.Leu826Pro; c.2891G>C, p.Arg964Pro; c.3207C>A, p.Tyr1069X; c.3364delT, p.Ser1122fs; c.3717T>G, p.Tyr1293X; c.3871G>A, p.Ala1291Thr; c.4306_4312del, p.Thr1436fs) were found in seven unrelated patients. Currently, our mutation detection score is at least one *ABCC6* mutation in 87% of patients with a clinical diagnosis of PXE.

**Conclusions:**

Our results support that *ABCC6* is the most important, and probably the only, causative gene of PXE. In total, 188 different *ABCC6* mutations have now been reported in PXE in the literature.

## Introduction

Pseudoxanthoma elasticum (PXE; OMIM 264800) is a heritable disorder of connective tissue, affecting the skin, retina, and blood vessels. The most frequent retinal abnormalities are peau d’orange, angioid streaks, and punched-out white chorioretinal lesions, which are visible as white dots sometimes with a comet-like tail. Angioid streaks develop in almost all of the patients and often lead to choroidal neovascularization, subretinal hemorrhages, and visual loss. Skin abnormalities usually start at the lateral sides of the neck with yellowish papules that confluence into plaques. Skin of other flexural sides of the body often follows the same course, and sometimes the skin abnormalities progress to redundant folds. Patients have an increased risk of cardiovascular complications including gastro-intestinal bleedings and arteriosclerosis. The expression of the disease is variable, and substantial clinical differences exist between patients, even within families [1,2].

PXE is caused by mutations in a single gene, *ABCC6* [3-5]. The inheritance is autosomal recessive. Recent clinical and molecular studies showed that putative dominant segregating PXE pedigrees are probably the result of mild expressions in heterozygous carriers or pseudodominance due to an unexpected high carrier frequency in the population [6-9].

**Figure 1:**
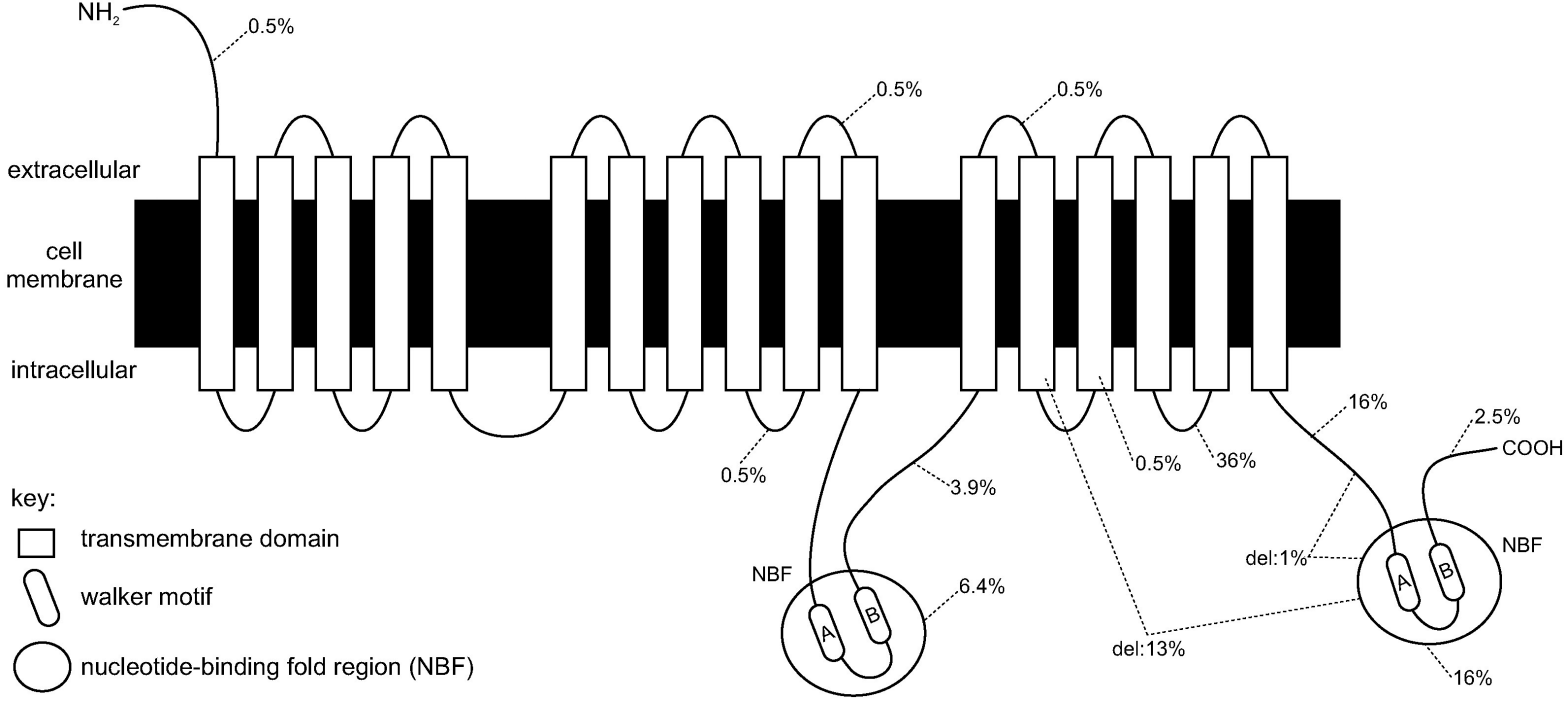
Schematic representation of the MRP6 protein. The protein contains 17 membrane-spanning domains and two intracellular nucleotide binding folds (NBFs). The percentages in the figure show how the mutations in our population are distributed over the different domains of the protein. The eighth cytoplasmatic loop is the most frequently mutated domain, followed by the region between the last transmembrane-spanning domain and NBF2 and by NBF2 itself. Del=deletion. In addition, 1% of the population had a deletion of the whole gene.

*ABCC6* belongs to the ATP-binding cassette (ABC) gene sub-family C [10,11]. *ABCC6* has 31 exons spanning about 73 kb genomic DNA. The mRNA is approximately 6 kb with an open reading frame (ORF) of 4.5 kb. The ABCC6 protein consists of 1,503 amino acids and contains 17 transmembrane-spanning domains and two intracellular nucleotide binding folds (NBFs). The NBFs consist of Walker A and B domains and a C motif critical for active ATP dependent transport across the cell membrane [1,12]. The putative ABCC6 protein structure is presented in Figure 1. Two *ABCC6* pseudogenes, homologous to, respectively, the first four and nine exons of *ABCC6*, have been identified [13,14], complicating the mutational analysis of the gene [15].

*ABCC6* is highly expressed in the liver and kidney. Expression in multiple other tissues including those affected by PXE (skin, retina, and vessel walls) is low or absent. The protein was localized to the basolateral membranes of both hepatocytes in the liver and the proximal kidney tubules. The natural substrate transported by the ABCC6 protein remains to be identified. Functional studies showed that ABCC6 transports glutathione S-conjugate leukotriene C(4), S-(2,4-dinitrophenyl) glutathione, and the cyclopentapeptide BQ123 [16-18]. The present view is that ABCC6 transports substrates from the liver and kidney cells back into the blood [10,18].

Up to now, 180 different mutations in *ABCC6* have been reported (Appendix 1). Mutations were found throughout the gene, but there is a high concentration in and around the NBFs and in the eighth cytoplasmatic loop [7,9,12,19-21]. Mutation detection rates varied from 55%-97% among the different studies.

The purpose of this paper was to report eight novel mutations in *ABCC6* and to update the mutation spectrum and frequency of *ABCC6* mutations in PXE patients.

## Methods

DNA of all probands/families (n=166) was collected by one of us or sent to the Netherlands Institute for Neuroscience, a referral center for geneticists and ophthalmologists from the Netherlands and other European countries, for mutational analysis of *ABCC6*. The patients, who had novel mutations, were clinically examined by one of us (A.P., B.C., H.K., A.M., P.dJ.) except for cases 4 and 7, and data from dermatologic, ophthalmic, and cardiovascular examinations elsewhere were collected. The dermatologic examination consisted of an inspection of the skin and histopathology of a skin biopsy. The ophthalmic examination included an assessment of visual acuity, slit-lamp examination of the anterior segment, biomicroscopy with a 90 diopter lens of the posterior pole of the eye fundus, indirect ophthalmoscopy of the peripheral retina, and digital photography of as many of the fundus signs as feasible. The cardiovascular examination included at least measurement of blood pressure, electrocardiography, and echocardiography. The clinical diagnosis of PXE was considered definite if at least two of the three following criteria were met: characteristic skin lesions at the lateral side of the neck and/or other flexural regions of the body, fragmentation and calcification of elastic fibers in a skin biopsy, and characteristic retinal lesions (peau d’orange, angioid streaks, and/or punched-out white chorioretinal lesions).

DNA was isolated from peripheral blood by standard techniques. Polymerase chain reaction (PCR) primers, amplification conditions, and mutation analysis strategy were essentially performed as described previously [22]. After pre-screening for common mutations, all coding exons were screened by denaturing high-performance liquid chromatography (dHPLC). Exonic fragments with changed dHPLC patterns were further analyzed by direct sequencing. The known deletions of exons 23–29 and exon 15 were analyzed as described [22].

The nomenclature for mutations was based on previously published recommendations [23] and additional guidelines. The *ABCC6* cDNA consensus sequence (GenBank AF076622) was used for DNA mutation description.

When a novel mutation was found, at least 140 control chromosomes were screened for this mutation. The controls were Caucasian individuals without any eye disease or any other apparent disorder. When the mutation was a missense mutation, conservation of the changed amino acid was checked by ClustalW multiple sequence alignment comparison for ABCC6 or other closely related proteins in *Felis catus, Gallus gallus, Monodelphis domestica, Mus musculus, Ornythorhynchus anatinus, Otolemur garnetii, Pan troglodytes*, and *Rattus norvegicus*. An amino acid was considered to be conserved when it was present in multiple proteins in these animals.

## Results

**Figure 2:**
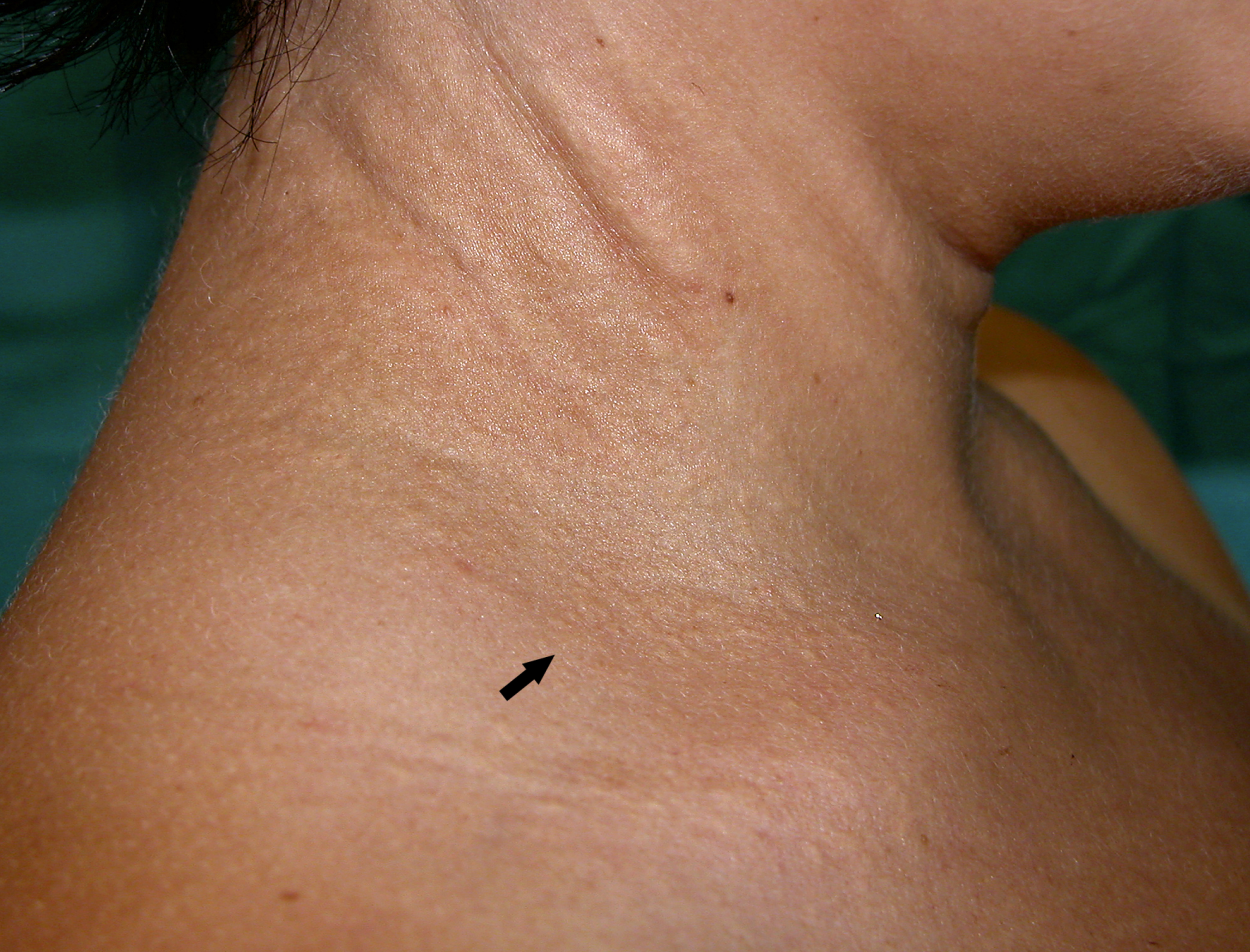
Clinical skin features of pseudoxanthoma elasticum. Mild but characteristic skin features, consisting of papules (as indicated by an arrow) and redundant skin folds as shown on the right side of the neck of case 1. In a later stage the papules can confluence into plaques.

We found eight novel *ABCC6* mutations (c.1685T>C, c.2477T>C, c.2891G>C, c.3207C>A, c.3364delT, c.3717T>G, c.3871G>A, and c.4306_4312del). None of these mutations were present in the control chromosomes. The eight novel mutations were found in seven patients in whom the clinical diagnosis of PXE was unambiguously established. All had characteristic skin abnormalities (Figure 2) either confirmed by a skin biopsy or in combination with characteristic ophthalmologic signs or by both (Table 1). Below, we present the molecular data in detail together with family data where relevant.

**Table 1:** Summary of the demographic and clinical data of the patients with novel mutations

Case	Allele 1	Allele 2	Sex	Age (years)	Origin	Age of onset	Skin	Biopsy	Eyes	CV	Fam
1	c.3662G>A	**c.1685T>C**	F	28	E	16	+ (Figure 2)	+	as	n	neg
2	c.2787+1G>T	**c.2477T>C**	M	13	GB	10	+	+	n	n	neg
3	**c.2891G>C**	-	F	31	GB	29	+	nd	as	n	?
4	**c.3207C>A**	**c.3871G>A**	M	36	NL	?	+	+	?	?	?
5	del exon 23–29	**c.3364delT**	F	29	NL	29	+	+	pdo, as (Figure 3)	?	pos
6	**c.3717T>G**	**c.3717T>G**	F	25	TR	19	+	+	pdo, as, co	mi	pos
7	c.4015C>T	**c.4306_4312del**	F	43	S	?	+	?	as	n	neg

The diagnosis of pseudoxanthoma elasticum (PXE) can be made if a case has at least two of the following criteria: characteristic skin lesions, characteristic abnormalities in a skin biopsy, characteristic retinal lesions. Based on these criteria, all of our cases have a definite diagnosis of PXE. No genotype-phenotype correlations can be made in this small group. +=affected, as=angioid streaks, co=comet-like lesions, cv=cardiovascular, E=Spain, F=female, fam=family history, GB=Great Britain, M=male, mi=mitral valve insufficiency, n=normal, nd=not done, neg=negative, NL=Netherlands, pdo=peau d’orange, pos=positive, S=Sweden, TR=Turkey, yrs=years, ?=no information, novel mutations are bold and underlined.

In case 1, analysis of *ABCC6* revealed two mutations, the earlier reported c.3662G>A (p.Arg1221His) [24] and the novel mutation c.1685T>C (Met562Thr). This latter missense mutation changes a well conserved amino acid at the fifth extracellular loop of the protein.

In case 2, we found the previously reported pathogenic sequence change, c.2787+1G>T, in intron 21 of *ABCC6* [25,26] and the novel mutation, c.2477T>C (p.Leu826Pro), in exon 19. This missense mutation is located just after NBF1 and leads to the change of a well conserved amino acid.

In case 3, one missense mutation was found in *ABCC6*, the novel mutation c.2891G>C (p.Arg964Pro), which leads to the change of a well conserved amino acid between NBF1 and NBF2. No second mutation was found.

In case 4, we found a c.3207 C>A (p.Tyr1069X) nonsense mutation as well as a c.3871G>A (p.Ala1291Thr) missense mutation in *ABCC6*. Both mutations have not been reported before. The former mutation theoretically results in a premature chain termination and an absent or dysfunctional protein. The latter mutation changes a well conserved amino acid in NBF2.

**Figure 3:**
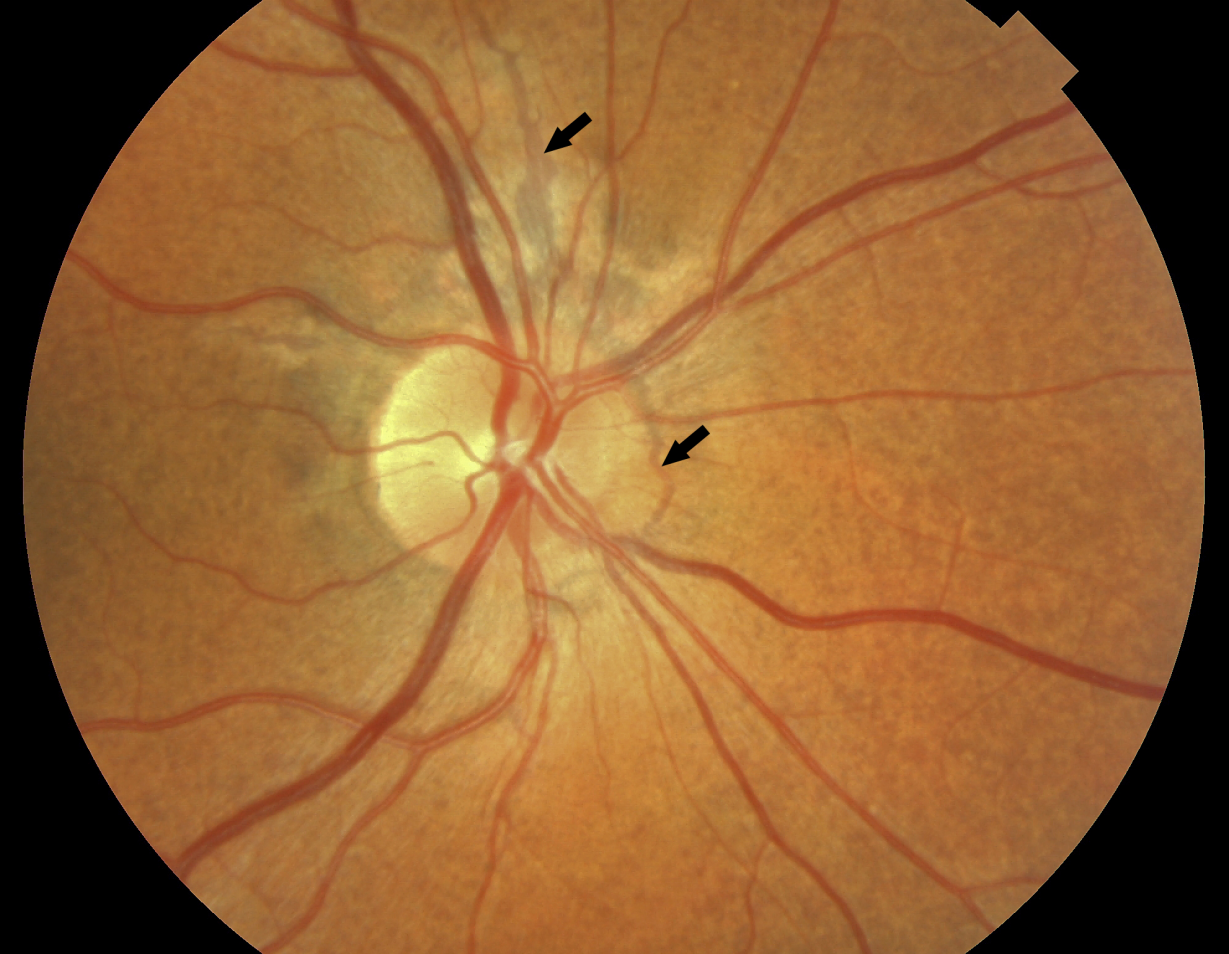
Clinical optical features of pseudoxanthoma elasticum. 141514The retina of the right eye of case 5 shows peau d'orange (diffuse, mottled hyperpigmentation) and angioid streaks, as indicated by arrows. The angioid streaks resemble retinal blood vessels and radiate from the optic disc to the periphery of the retina. These two signs are the most frequent ophthalmologic features of pseudoxanthoma elasticum.

In case 5, two mutations were found in *ABCC6*, the well-known deletion of exon 23–29 and the novel mutation, c.3364delT. The angioid streaks and peau d’orange of the retina of her right eye can be observed in Figure 3. She had minimal skin abnormalities at the lateral side of her neck, just below the hair line, which were not noticed before. Her father also had a clinical diagnosis of PXE. He had peau d’orange of the retina, angioid streaks, and macular degeneration after subretinal neovascularization in both eyes. The dermatologist did not find characteristic skin lesions, but a skin biopsy from the left side of the neck showed clumping and calcification of elastic fibers. He had the exon 23–29 deletion. The mother of case 5 had the c.3364delT mutation. We did not find a second mutation in the father.

Case 6 was homozygous for a novel mutation in *ABCC6*, c.3717T>G leading to a stop codon at position 1239 of the protein (p.Tyr1239X) just before NBF2. The patient’s sister was said to be affected as well. Consanguinity between the parents was denied.

Mutational analysis of *ABCC6* in case 7 revealed the earlier reported mutation, c.4015C>T (p.Arg1339Cys) [25], and the novel mutation, c.4306_4312del. This new mutation theoretically leads to a frameshift and the introduction of a stop codon at position 1461 of the protein.

In our entire data set, we found two causative *ABCC6* mutations in 76 of 166 (46%) PXE probands and only a single mutation in 51 (31%) probands. In 39 (23%) probands, we did not find any mutation. PXE had been diagnosed in 19 of these patients. In the remaining 20 patients, there was either doubt about the diagnosis or not enough clinical data to prove the diagnosis. Thus at least one mutation was found in 127 (87%) of 146 probands in whom the clinical diagnosis was established a priori. This means that we found mutations in 203 (69.5%) out of 292 alleles. The type and frequencies of the different mutations found are listed in Appendix 1. Part of the patient and mutation data described here was published previously [19,22].

## Discussion

In this study, eight novel mutations were found, which we consider to be implicated in PXE: c.1685T>C (p.Met562Thr), c.2477T>C (p.Leu826Pro), c.2891G>C (p.Arg964Pro), c.3207C>A (p.Tyr1069X), c.3364delT (p.Ser1122fs), c.3717T>G (p.Tyr1293X), c.3871G>A (p.Ala1291Thr), and c.4306_4312del (p.Thr1436fs). All of these mutations were not present in at least 140 control chromosomes, and the missense mutations lead to the change of a well conserved amino acid. This suggests that these mutations are pathogenic. Four novel mutations target the eighth cytoplasmatic loop (c.3364delT) or NBF2 (c.3717T>G, c.3871G>A, and c.4306_4312del). Both domains are known hot spots for *ABCC6* mutations. Three other new mutations (c.2477T>C, c.3207C>A, and c.2891G>C) are located in between NBF1 and NBF2. The c.2477T>C mutation occurs just after NBF1 in an area targeted by three previously described mutations (c.2420G>A, c.2428G>A, and c.2458G>C). The c.3207C>A and c.2891G>C mutations target less frequently mutated domains of the ABCC6 protein. The last novel mutation, c.1685T>C, is uniquely located at the fifth extracellular loop before NBF1.

In our entire data set, at least one *ABCC6* mutation was identified in 127 (77%) of 166 probands in which there were clinical suspicions of PXE. If we exclude the 20 patients whose clinical diagnoses were questionable, we find at least one mutation in 87% of probands. We found 40 different mutations of which c.3421C>T (p.Arg1141X), c.3775delT, and a deletion of exons 23-29 were most frequent. The p.Arg1141X mutation was found in 33% of alleles with a mutation, and the latter two mutations were found in 14% and 13% of the alleles, respectively.

We did not find any mutation in 19 of the 146 (13%) probands with a clinical diagnosis of PXE. In 51 of 146 (35%) probands, we only found one mutation. As inheritance is autosomal recessive, it is to be expected that these probands have two mutations. Taken together, our missing rate per allele is 35%, which suggests that a considerable part of the mutations in our data set could not be identified.

Four studies in which the patients had clinically definite PXE diagnoses reported missing rates of 3% [7] or 17.1% [27] after sequencing and 12.3% [20], 14% [28], or 34% [21] after denaturing high-performance liquid chromatography (dHPLC). It is largely unclear why mutation detection rates are different in several studies. What are the possible explanations?

### Geographic differences in patient populations

Most of the patients in the above mentioned studies were from Italy [27], Germany [20], France [28], and the United States [7,21]. Most of our patients were from the Netherlands. Mutation frequencies differ in different populations [12]. It is conceivable that a relatively frequent mutation in certain populations can be missed by the techniques used. Heterozygous deletions of (or part of) the gene and mutations in the promoter region and in introns can easily be missed. Thirteen French PXE patients with at least one unidentified mutation (18 unidentified alleles) after dHPLC were studied with a quantitative multiplex PCR of short fluorescent fragments (QMPSF) [28]. Five (novel) deletions were detected. This reduced the total missing rate from 14% to 10% of 130 alleles. Deletions can also be detected with multiplex ligation-dependent probe amplification (MLPA), but both QMPSF and MLPA are not yet routinely used.

### Sequencing

Sequencing the gene will yield additional missense mutations, which are missed by dHPLC. This could at least partly explain the high detection rate of Miksch et al. [7].

### Differences in patient selection

We did not have detailed clinical information of all patients and, as a result, there were some patients without a definite diagnosis of PXE. Some patients could have a PXE-like phenotype as can be seen in beta-thalassemia, sickle cell anemia, and peri-umbilical perforating PXE [1].

### Digenic inheritance

Another, less likely, possibility is digenic inheritance in the patients without two mutations. The combination of one *ABCC6* mutation with a mutation in another gene could lead to PXE. This could be different for different populations.

Including the mutations presented here, at least 188 different *ABCC6* mutations have been published to date in the international literature (Appendix 1). In the European populations, the p.Arg1141X mutation is by far the most prevalent (about 28% of alleles) while in the United States population, the exon 23–29 deletion occurs most frequently (also about 28% of alleles) [25,29]. The mutation distribution in *ABCC6* (Figure 1) shows three mutation hot spot domains: the first and second NBF as well as the eighth cytoplasmatic loop. Indeed, the latter is truly a hot spot for mutations since the frequent mutations, p.Arg1141X and the exon 23–29 deletion, target this area. It has been suggested that the eighth cytoplasmatic loop may be involved in ABCC6 substrate recognition [12]. In our data set, mutations in these three domains were found in 71% of the alleles with mutations. Screening of the involved exons 16–18, 24, and 27–30 together with the detection of the exon 23–29 deletion would detect 95% of all of our mutations.

In all of our patients who had novel mutations, a diagnosis of PXE could be made clinically (Table 1). The group was too small to establish genotype-phenotype correlations. In the data set, we observed considerable intra-and interfamilial variations in phenotype, and we could not extract a genotype-phenotype relationship either. Previous reports on potential genotype-phenotype correlations revealed variable results. Diagnosis at a significantly younger age and a higher number of affected organs were found in the case of mutations that lead to an absence of (functional) MRP6 [30]. It was suggested that nonsense mutations were more frequently associated with generalized involvement [27]. However, even in an extended patient series, no clear genotype-phenotype correlation could be found to date [7,19,21,25,27]. Besides, there is marked variable expression within family members with the same genotype [19,27,31].

In summary, we provide further evidence that *ABCC6* is the most important, and probably the only, causative gene implicated in PXE. We added eight new mutations to the *ABCC6* mutation spectrum and supported the notion that most mutations are present in the cytoplasmatic domains at the carboxy terminal end of the protein, especially in the three putative important functional domains of ABCC6 (NBF1, NBF2, and the eighth cytoplasmatic loop).

## Appendix 1. All *ABCC6* mutations which are implicated in pseudoxanthoma elasticum

To access the data, click or select the words “Appendix 1”. This will initiate the download of a compressed (zip) archive that contains the file. This file should be uncompressed with an appropriate program (the particular program will depend on your operating system). We found mutations in 203 alleles. The fourth column shows the frequency of the different mutations among the alleles. Novel mutations are bold and underlined.

References found only in Appendix:

32. Struk B, Cai L, Zach S, Ji W, Chung J, Lumsden A, Stumm M, Huber M, Schaen L, Kim CA, Goldsmith LA, Viljoen D, Figuera LE, Fuchs W, Munier F, Ramesar R, Hohl D, Richards R, Neldner KH, Lindpaintner K. Mutations of the gene encoding the transmembrane transporter protein ABC-C6 cause pseudoxanthoma elasticum. J Mol Med 2000; 78:282–6.10954200

33. Yoshida S, Honda M, Yoshida A, Nakao S, Goto Y, Nakamura T, Fujisawa K, Ishibashi T. Novel mutation in ABCC6 gene in a Japanese pedigree with pseudoxanthoma elasticum and retinitis pigmentosa. Eye 2005; 19:215–7.15184964

34. Meloni I, Rubegni P, De Aloe G, Bruttini M, Pianigiani E, Cusano R, Seri M, Mondillo S, Federico A, Bardelli AM, Andreassi L, Fimiani M, Renieri A. Pseudoxanthoma elasticum: Point mutations in the ABCC6 gene and a large deletion including also ABCC1 and MYH11. Hum Mutat 2001; 18:85.11439001

35. Ringpfeil F, Nakano A, Uitto J, Pulkkinen L. Compound heterozygosity for a recurrent 16.5-kb Alu-mediated deletion mutation and single-base-pair substitutions in the ABCC6 gene results in pseudoxanthoma elasticum. Am J Hum Genet 2001; 68:642–52.11179012

36. Katona E, Aslanidis C, Remenyik E, Csikos M, Karpati S, Paragh G, Schmitz G. Identification of a novel deletion in the ABCC6 gene leading to Pseudoxanthoma elasticum. J Dermatol Sci 2005; 40:115–21.16183260
